# Development of a multi-gene-based immune prognostic signature in ovarian Cancer

**DOI:** 10.1186/s13048-021-00766-4

**Published:** 2021-01-28

**Authors:** Tiefeng Cao, Huimin Shen

**Affiliations:** Department of Gynecology and Obstetrics, Sun Yat-Sen University, First Affiliated Hospital, 58 zhongshan 2nd road, Yuexiu District, Guangzhou, Guangdong 510070 P.R. China

**Keywords:** Ovarian Cancer, Immunogenic landscape, Prognostic signature, Transcription factors, Personalized therapy

## Abstract

**Background:**

Various components of the immune system play a critical role in the prognosis and treatment response in ovarian cancer (OC). Immunotherapy has been recognized as a hallmark of cancer but the effect is contradictional. Reliable immune gene-based prognostic biomarkers or regulatory factors are necessary to be systematically explored to develop an individualized prediction signature.

**Methods:**

This study systematically explored the gene expression profiles in patients with ovarian cancer from RNA-seq data set for The Cancer Genome Atlas (TCGA). Differentially expressed immune genes and transcription factors (TFs) were identified using the collected immune genes from ImmPort dataset and TFs from Cistoma database. Survival associated immune genes and TFs were identified in terms of overall survival. The prognostic signature was developed based on survival associated immune genes with LASSO (Least absolute shrinkage and selection operator) Cox regression analysis. Further, we performed network analysis to uncover the potential regulators of immune-related genes with the help of computational biology.

**Results:**

The prognostic signature, a weighted combination of the 21 immune-related genes, performed moderately in survival prediction with AUC was 0.746, 0.735, and 0.749 for 1, 3, and 5 year overall survival, respectively. Network analysis uncovered the regulatory role of TFs in immune genes. Intriguingly, the prognostic signature reflected the immune cells landscape and infiltration of some immune cell subtypes.

**Conclusions:**

We first constructed a signature with 21 immune genes of clinical significance, which showed promising predictive value in the surveillance, and prognosis of OC patients.

**Supplementary Information:**

The online version contains supplementary material available at 10.1186/s13048-021-00766-4.

## Introduction

Ovarian cancer (OC) causes the most deaths among gynecological cancers, with more than 22,000 new cases and 14,000 deaths each year in the United States [1]. It is challenging that the incidence, the recurrence rate and chemotherapy-resistant cases is greatly increased despite the development of aggressive frontline treatment.

Evidence has shown that OC is immunogenic [2]. Immunotherapy has shown to be correlated with improved clinical outcome. Hamanishi et al. reported that the overall response rate for nivolumab treatment was 15% and the disease control rate is 45% [3]. 2015 ASCO annual meeting presented that pembrolizumab (anti-PD-1) and avelumab (anti-PD-L1) can be used as the immune checkpoint targets in clinical practice. Clinical trials, NCT02718417 (Javelin Ovarian 100), ENGOT-ov29-GCIG (ATALANTE), NCT02580058 (Javelin Ovarian 200), and NRG-GY009 as indicated, are ongoing or planned for the testing of potential efficacy of immunotherapy. However, ovarian cancer is featured with high clonal heterogeneity and specific dissemination patterns. Single chemotherapy or immunotherapy was less effictive, while combinatorial therapy may increase the risk of adverse effects [4]. New biomarkers or the regulators of immune system should be developed. Recent genome-wide studies addressed the impact of diverse gene regulatory mechanisms in immune homeostasis. Emerging evidence showed that transcriptional networks drive functional changes during immune activation and subsequent immune resolution. Thus, finding the neo-antigens or effective biomarkers, and identification of transcriptional regulators in the immune system and the regulatory networks between immune genes and transcriptional factors in the microenvironment is critical to improve the clinical outcome. The current study aimed to identify the immune genes correlated with the clinical prognosis in ovarian patients, and to develop and validate an individualized prognostic signature based on immune-related genes. Bioinformatics analysis was conducted based on the combined transcriptomes and immune gene profiling data from 376 ovarian cancer patients in TCGA and 88 normal ovarian tissues in GTEx (Genotype-Tissue Expression) dataset. A prognostic signature based on 21 immune genes was identified, and it is closely related with aggressive clinical outcomes of OC. Moreover, network analysis showed the close association between transcription factors and immune genes, thus describing the regulatory network of the immune landscape in the microenvironment.

## Materials and methods

### Data collection and preprocessing

The work flow is shown in Fig. S1. RNA-Seq data as well as clinical information was downloaded from TCGA dataset including 379 serous ovarian cancer cases, and three overlapping samples (TCGA.13.1489.02A, TCGA. 29.2414.02A, TCGA.61.2008.02A) were removed. RNA-Seq data and clinical information for 88 normal ovarian samples were obtained from GTEx in xena (// xenabrowser.net/). GEO (Gene Expression Omnibus) datasets, with accession number GSE26712 and GSE63885 based on GPL96 [HG-U133A] Affymetrix Human Genome U133A Array were downloaded for validation datasets. Clinical and pathologic characteristics of cases included was summarized in Appendix Table 1. Gene expression level was defined as the average value for multiple probes. All statistics were under R condition, and NormalizeBetweenArrays was used to normalize expression distribution.

### Differentially expressed immune gene (DEIGs)

1811 immune related genes were downloaded from ImmPort database (https://immport.niaid.nih.gov) including genes related to cytokines, T-cell signaling pathway, B-cell signaling pathway, NK (natural killer) cells signaling pathway etc. RNA-Seq data in TCGA and GTEx were used to identify the DEIGs between serous ovarian cancers and normal cases. The *P*-value thresholds were established by Bonferroni-correction method, which set the significance level to be 0.05 divided by number of tests. Consequently, DEIGs were selected by *p*-value < 2.76 × 10^− 5^ (0.05/1811) and absolute fold change > 2. The R package “Limma” was used to find out differentially expressed immune genes.

### Immune-gene based prognostic signature construction

First, survival-associated immune genes (SAIGs) were selected using the univariate Cox regression analysis in terms of overall survival (OS) of patients . LASSO (Least absolute shrinkage and selection operator) Cox regression analysis, by constructing a penalty function, was used to identify the predictive genes and construct the multi-gene-based prognostic model. Based on the expression level of each gene and the regression coefficient, we conducted the gene signature with the risk score (RS) (RS = ${\sum}_{i=1}^k{\beta}_i{Exp}_i$, *Exp*_*i*_ represents the expression level of each gene involved in the model and β_*i*_ represents the corresponding regression coefficient). Based on the median of RS, patients were divided into high-risk and low-risk groups. The Kaplan-Meier (K-M) survival analysis was used to compare the survival outcome between groups, and the ROC (receiver operating characteristic) curve was performed to determine the prediction value of this prognostic model with area under curve (AUC). Additionally, multivariable Cox regression analysis was used to define the independent prediction value of risk score from other prognostic factors including age, grade, stage, and debulking status. GSE26712 and GSE63885 datasets were used to validate the prediction value of the prognostic model.

### Transcription factors - immune genes regulatory network

318 transcription factors were obtained from Cistoma database (http://cistrome.org/). Differentially expressed transcription genes (DETGs) were identified with *p* < 1.57 × 10^− 4^ (0.05/318, Bonferroni-correction method) and absolute fold change > 2. We analyzed the correlation between 318 DETGs and 71 SAIGs, and constructed the regulatory network with the following condition: *P*-value < 2.21 × 10^− 6^ (0.05/(318*71), Bonferroni-correction method) and Pearson correlation coefficient > 0.3. Cytoscape software was used to visualize the network.

### Correlation between immune genes and clinical features

To evaluate the correlation between immune genes and clinical features including age, grade and stage, we compared the risk score of patients with age<60 or age ≥ 60, stage I or stage II or stage III in high grade serous ovarian cancer. Student’s t-test (for binary clinical variables) and ruskal Wallis test (for multiple clinical variables) were used.

### Correlation between immune genes and immune cells

CIBERSORT algorithm, namely gene expression deconvolution Algorithm, was used to evaluate the relative abundance of 22 kinds of immune cell with normalized gene expression data. It outperforms previous deconvolution methods with respect to noise, unknown mixture content, and closely related cell types. The 22 cell types inferred by CIBERSORT encompass T cells, B cells, natural killer cells, macrophages, dendritic cells, and neutrophils, amongst others. We uploaded TCGA RNA-Seq data to the CIBERSORT web portal (http://cibersort.stanford.edu/), and set the default signature matrix as 1000 permutations. We select samples with CIBERSORT-*P* value < 0.05 for further analysis. Correlation analysis was conducted to estimate the relationship between risk score and immune cells.

### Functional annotation and analysis

To show the biological understanding of the SAIGs, enrichment analysis was performed with DAVID (Database for Annotation, Visualization and Integrated Discovery) Bioinformatics Resources (version 6.8; https://david.ncifcrf.gov/). The 71 survival associated immune genes were uploaded and the biological processed of gene ontology with *P* < 0.05 were examined.

## Results

### The prognostic signature is established with 21 immune-based genes

**Fig. 1:**
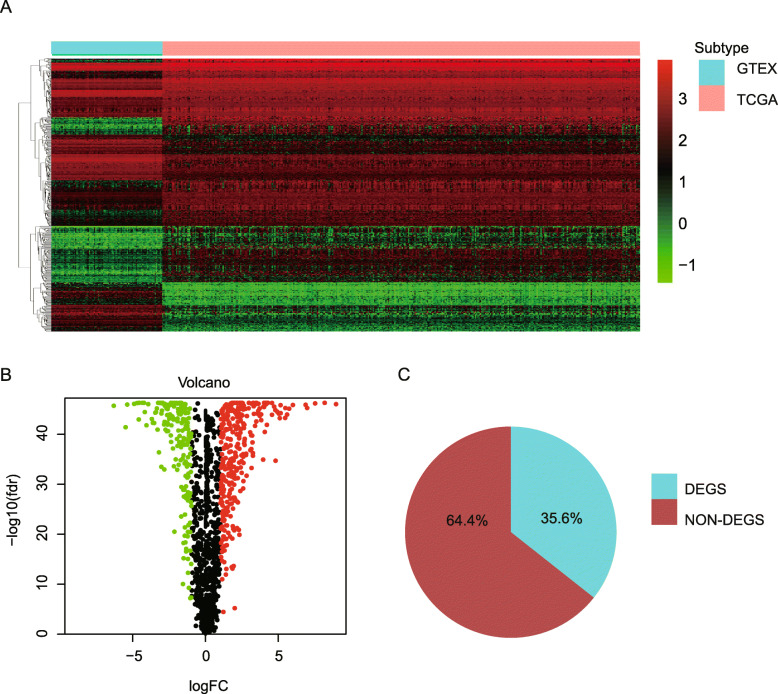
DEIGs between ovarian cancer and normal cases. **a** Heat map of the DEIGs. The above horizontal axis shows the information of samples including normal cases (*N* = 88) and ovarian cancer cases (*N* = 376), respectively. The left longitudinal axis shows the clustering results. The color change from red to green represents the expression of immune genes changed from high to low. **b** Volcano Plot of the DEIGs. The red and blue points in the figure show the DEIGs with statistical significant (*p*-value < 2.76 × 10^− 5^ and absolute fold change > 2). (C) DEIGs among 1811 immune related genes downloaded from the ImmPort database, with *p*-value < 2.76 × 10–5 and absolute fold change > 2. DEIG, differential expression immune genes

**Fig. 2:**
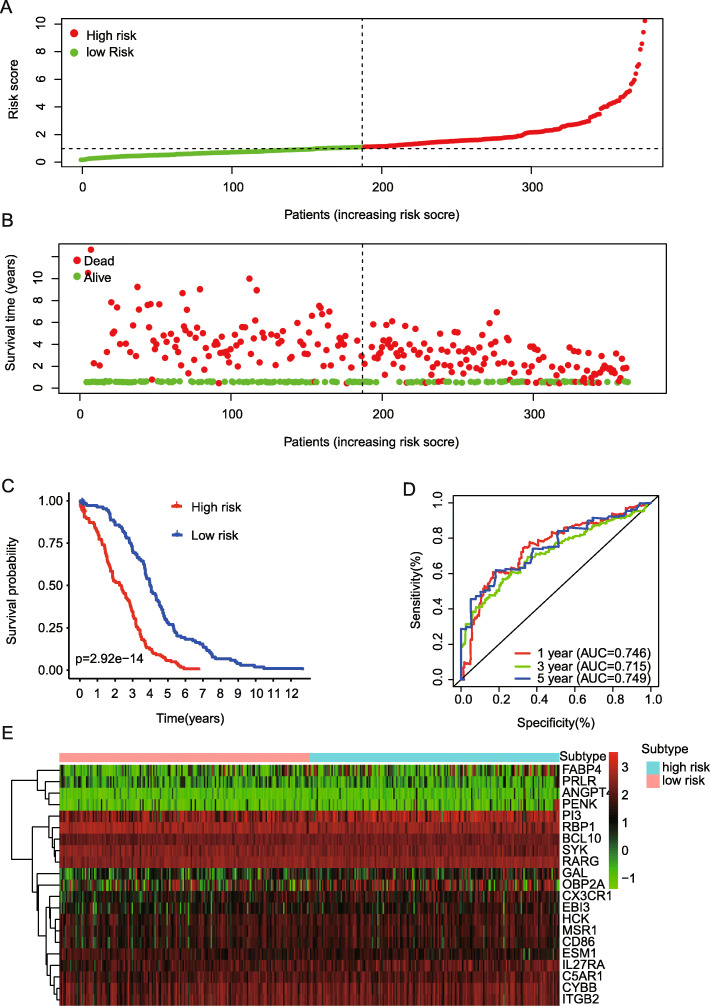
Prognostic signature Construction. **a** & **b** Patients with higher risk score in this model predicted poor prognostic survival and clinical outcome. **c** Kaplan-Meier curve of prognostic predictors for ovarian cancer. **d** ROC curves of prognostic predictors for ovarian cancer with 1 year, 3 year, and 5 year overall survival. **e** The expression distribution of the 21 hub genes in the prognostic model. LASSO, Least absolute shrinkage and selection operator; ROC, Receiver operating characteristic

A total of 376 ovarian cancer cases and 88 normal ovarian tissues were included in the eanalysis. Among 1811 immune related genes downloaded from the ImmPort database, as shown in Fig. 1a-c, a total of 495 differentially expressed genes were identified with *p*-value < 2.76 × 10^− 5^ and absolute fold change > 2, containing 188 downregulated and 307 upregulated genes (Table S1). 71 survival-associated immune genes with *p*-value < 0.05 by univariate COX analysis were identified among 495 differentially expressed genes in terms of overall survival (Fig. S2). We then constructed an prognostic model consisting of 21 hub genes among the top 40 survival-associated immune genes by ranking *p*-value from low to high from this set of genes by using LASSO cox regression (Fig. S3), and the 21 hub genes included IL27RA, GAL, RBP1, ANGPT4, EBI3, C5AR1, MSR1, HCK, SYK, CYBB, PI3, CD86, FABP4, CX3CR1, ITGB2, PENK, PRLR, RARG, ESM1, BCL10, and OBP2A. The risk score of 376 patients in TCGA dataset were defined with the 21 hub genes and the coefficient, as shown in Table S2. On the basis of the median value of risk score, we stratify patients into the high or the low immune risk groups in terms of overall survival (OS). As expected, higher risk score was related with poorer prognostic survival and clinical outcome (Fig. 2a & b), and the proposed model could successfully separate OC samples into high and low OS patients (Fig. 2c, *P* = 2.92e− 14). Moreover, the ROC curve analysis addressed that the prognostic signature had good prediction value for clinical outcome of ovarian cancer patients, with the AUC 0.746, 0.715, and 0.749 for 1 year, 3 year, and 5 year overall survival, respectively (Fig. 2d). Figure 2e showed the expression profiles pattern of these 21 hub genes in the final model.

### The prognostic signature is closely correlated with clinicopathological factors

The clinicopathological factors-based stratification analyses showed that the risk score is positively related with patients’ age and stage (Table S3). Comparing to the patients with age <60, elder patients had a higher risk score indicating poor clinical outcome(*P* = 0.019, Fig. S4A). In addition, the comparison of patients with stage II, III, IV showed that patients with advanced stage had a higher risk score (*P* = 0.048, FigS4B).

### The prognostic signature is an independent prognostic factor in OC patients

**Fig. 3:**
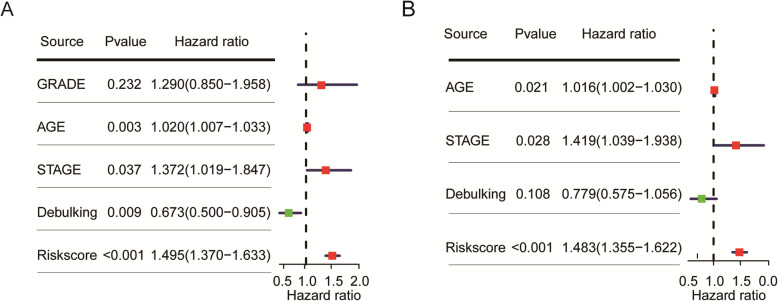
Univariate and Multivariable Cox regression survival analysis of prognostic signature adjusted for clinical factors in serous ovarian carcinoma. **a** Univariate Cox regression analysis, and **b**) Multivariable Cox regression analysis, showed that risk score was associated with poor overall survival and is the independent factor for clinical survival

As indicated in published articles, elder age, advanced stage, sub-optimal debulking status remained the poor prognosis factors in ovarian cancer. Our result by univariate Cox regression analysis showed the same association between these factors and overall survival, as well as higher risk score (Fig. 3a). In addition, the risk score remained as an independent prognostic factor after adjusting for clinical factors such as age, debulking status, and stage by applying the multivariate Cox regression analysis (HR = 1.483, 95%CI:1.355–1.622, *p* < 0.001, Fig. 3b).

### The prognostic signature has great prediction value in other two independent datasets

**Fig. 4:**
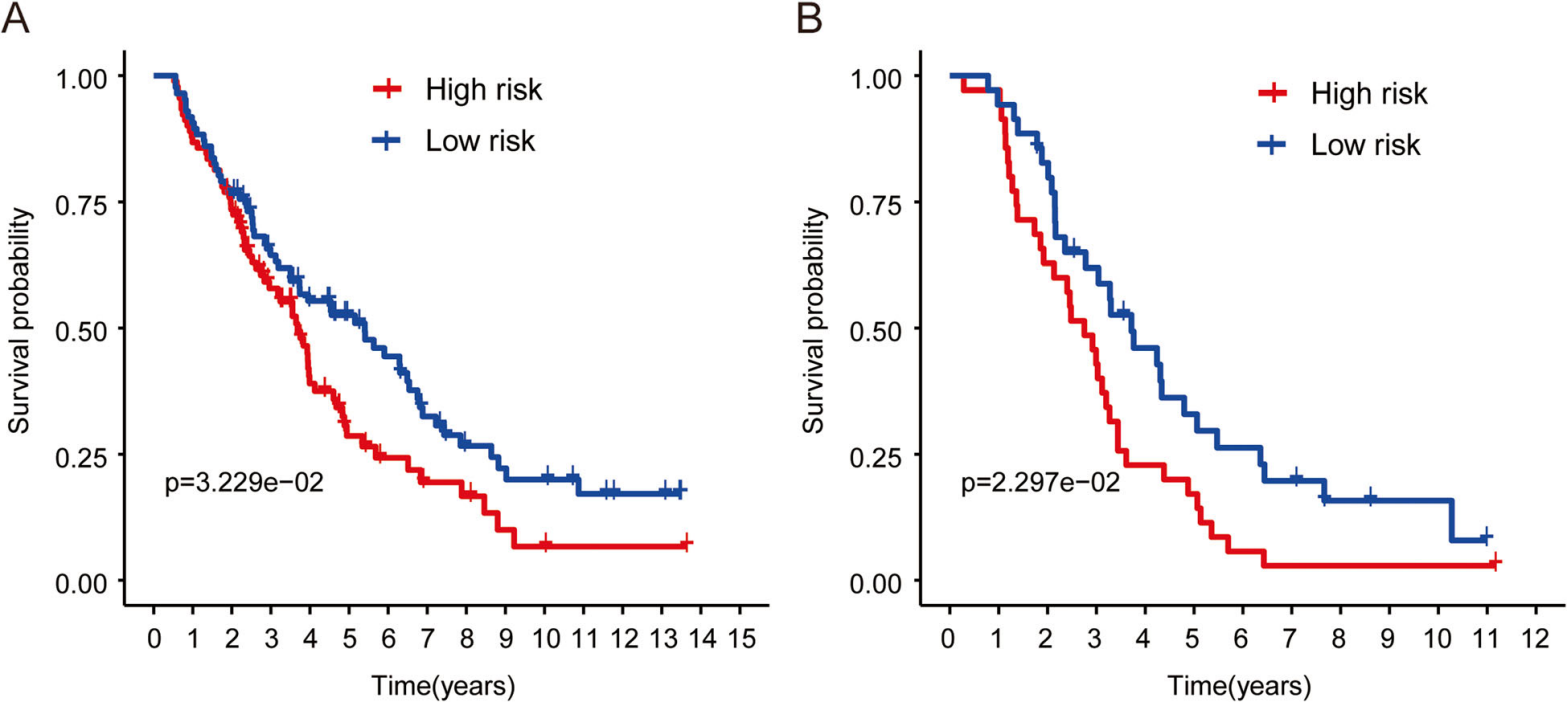
The prediction value of this prognostic signature with other two independent datasets. Validation showed that high-risk was correlated with poor prognosis in GSE26712 (**a**), and GSE63885 (**b**)

The gene profiling and clinical information of two independent groups, GSE26712 and GSE63885 datasets, were downloaded to further validate the prediction value of the model. The corresponding risk score of each patients were calculated with the constructed model above, and the patients were divided into high-risk and low-risk sub-groups by the medium of risk score. As expected, Kaplan-Meier curves illustrated that risk score is correlated with clinical prognosis, showing that patients in high-risk subgroup was correlated with poor prognosis when comparing with patients in low-risk group (Fig. 4a & b, *P* = 0.032 and *P* = 0.022, respectively).

### Immune gene-based prognostic signature is related with infiltration of specific immune cell subtypes

**Fig. 5:**
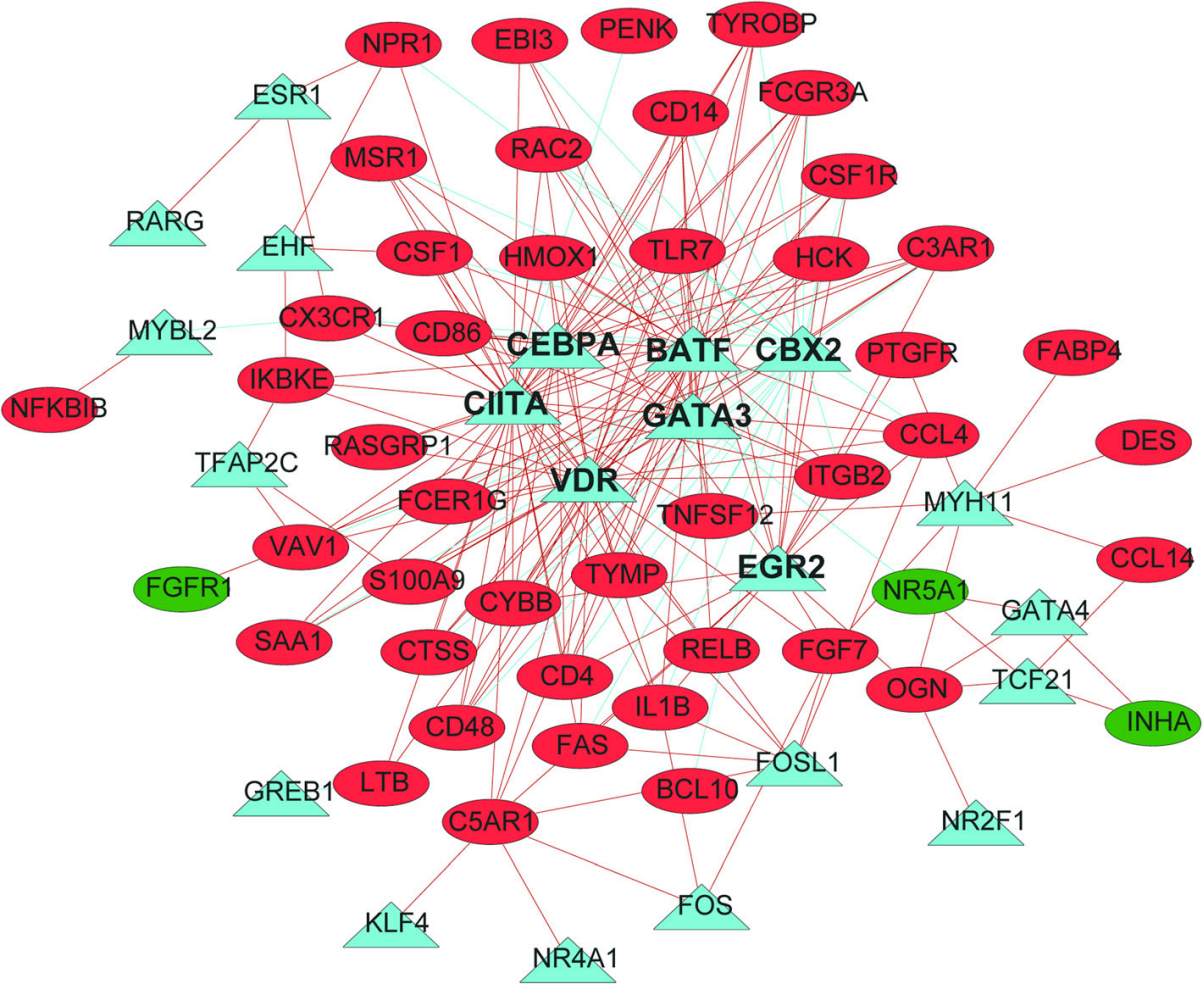
The correlation network analysis between survival-associated transcription factors and immune genes in serous ovarian cancer patients constructed by Cytoscape. Survival-associated immune genes (circle) positively (red lines) or negatively (green lines) correlated with transcription factors (triangle), which predicted good (green circle) or poor (red circle) clinical survival in serous ovarian cancer patients

The immune landscape presented with immune genes is mainly contributed by immune cells. To understand the correlation between immune gene signature and specific immune cell infiltrates, we performed CIBERSORT algorithm to determine the proportions of 22 immune cells infiltrates. As indicated, high density of Macrophages M0 (Cor = − 0.263, *p* = 2.235e− 04, FigS5A), NK cells resting (Cor = − 0.155, *p* = 0.032, FigS5B), and T cells follicular helper (Cor = − 0.163, *p* = 0.024, FigS5C) infiltration was negatively correlated with higher risk score, while Macrophages M2(Cor = 0.262, *p* = 2.356e− 04, FigS5D), Neutrophils (Cor = 0.176, *p* = 0.014, FigS5E), and T cells CD8(Cor = 0.171, *p* = 0.018, FigS5F) were positively correlated with higher risk score.

### Functional annotation of the immune gene-based signature

Enrichment analysis of the 71 survival associated immune genes was performed and we identified the biological processed in gene ontology (Fig. S6). As indicated, the inflammatory response is the most significant pathway involved. Intriguily, most biological processed were correlated with microenvironment including extracellular region, cytokine activity, angiogenesis etc.

### The prognostic signature is related with and regulated by transcription factors

The immune response is strictly controlled and regulated for the production of inflammatory cytokine. Transcription factors showed critical role in regulating gene expression, allowing immune response to occur in a controlled effective manner [5]. Besides, transcription factor can act as an immuno-metabolism regulator and control immune cell metabolism, playing an important role in the regulation of immune, malignant, and metabolic diseases [6]. Hence, to identify the interaction networks between transcription factors and immune genes is imperative in ovarian cancer. Firstly, 130 differentially expressed TFs were identified between ovarian cancer cases and normal tissues among 318 genes downloaded from Cistoma database (http://cistrome.org/) (Table S4). Regulatory network was built with 71 survival-associated immune genes and 130 TFs. As demonstrated in Fig. 5, there is close correlation between immune genes and transcription factors, showing that most of the survival-associated immune genes in the network are positively correlated with transcription factors. Specially, we identified four TFs, whose expression levels were significantly correlated with survival-associated immune genes, including CIITA, BATF, VDR, and CBX2. Furthermore, we searched the ENCODE dataset (https://www.encodeproject.org/) and GTRD databases (http://gtrd.biouml.org), and downloaded the ChIP-seq data of CIITA, BATF, VDR, and CBX2 that reflects the binding and regulatory effect directly. 28 immune related genes were identified among the 46 genes that we have shown to be correlated with TFs (Table S5). Thus, we addressed the regulatory network directly or indirectly between TFs and immune genes, which demonstrated the regulatory mechanism among these immune-related genes.

## Discussion

Immune related genes play a significant role in tumor progression and immunotherapy. An integrative, genome-wide profiling study to establish the immune gene-based signature to predict the clinical prognosis is urgently needed, and the molecular regulatory mechanisms of immune related genes and immune-tumor interation have not been identified. In the present study, we conducted comprehensive analysis and developed a prognostic signature based on 21 survival associated immune genes with the TCGA dataset as the training set and two independent GEO datasets as the validation set. Our immune-gene based prognostic signature can stratify clinical patients into high or low-risk subgroups with different clinical outcomes. We further leveraged the additional value of clinical and molecular features and showed that the immune genes could provide the valuable clinical biomarkers and regulated by transcription factors in serous ovarian cancer. The network analysis showed that immune genes were closely related with transcription factors, and this has been a critical regulatory mechanism for immune response.

Patients with ovarian cancer are at substantial risk for recurrence and chemotherapy resistant. Immunotherapeutic approaches such as personalized antigen-specific immunotherapy have been recognized as curative potential targets [7]. Currently, the immune-based interventions have gained approval in many solid tumors and hematologic malignancies. However, ovarian cancer has the features of extensive malignant and immunologic heterogeneity. New tumor antigens and prediction signature are critically needed to select cases that can benefit from the immune-based therapy. Previous studies demonstrated that next-generation sequencing or large-scale sequencing analysis is now available to identify the tumor neo-antigens for personalizing cancer immunotherapies [8], but they had the limitation of small sample size and inter-study heterogeneity [9]. Bioinformatics systematic analysis will enable a more in-depth exploration. In this study, we combined gene expression profiling from TCGA dataset, which had relative large samples with 376 cases, and GEO datasets. The 21 immune genes were identified as reliable biomarkers of ovarian cancer. Besides, exploration of immune gene patterns and survival-associated immune genes with computational biology that are specifically designed to perform analysis across different platforms can minimize the technical or samples bias, providing further and general insights into biomarkers identification. As such, this immune-gene based prognostic signature may serve as a generalized, individualized estimate of survival of ovarian cancer.

The responses of immunotherapy in ovarian cancer are variable with high cost for treatment. Prognostic or predictive biomarkers related with tumor immune microenvironment are urgently needed. Significant research on immune relevant prognostic signature proposed by Wen Jiang et al. aimed to find biomarkers predicting prognoses and immunotherapeutic responses in bladder cancer. Wen’s article only used the samples of patient to identify the differentially expressed genes associated with immune infiltration, while we used the cases including ovarian cancer patients and normal ones to identify the differentially expressed genes between cancer and normal cases. Thus, Wen constructed the tumor immune infiltration–associated gene (TIM) signature that can predict the immunotherapeutic response and reflect the immune cells infiltration, while the gene signature constructed in this article was based on survival-associated immune genes and can stratify ovarian cancer patients into two distinct subgroups related with survival outcomes. Combinatorial prognostic immune gene-based signature can illuminate how specific genomic aberration types associated with clinical outcome [10]. The correlation between the gene signature and prognostic factors provoked perspectives on the good predictive value of gene signature on distinct grade or stage disease and further on overall survival in OC.

However, immunotherapy can be prevented by tumor immunological function disruption, and the off-target activity of immune-stimulatory factors may result in severe toxicity. Individual immunotherapy is not efficiency for strong anti-tumor potential, while combinatorial immunotherapy may increase the risk and severity of adverse effects [4]. Thus, finding tumor-mediated immunosuppression or immunostimulation targets is still challenging [11]. Immunomodulatory gene circuit platform is potential for tumor-specific immune-stimulation by de novo cancer-specific promoter synthesis, with RNA-based design and transcription factors encoding. Differentiated TFs were identified, thus multiple binding motifs for cancer-specific TFs will beencoded to generate synthetic OC-specific promoters, resulting in compact and tumor-specific promoters [12]. It is promising that we can identify the specific TFs for promoters encoding. In the present study, we have demonstrated the interaction between transcription factors and immune genes, showing that the majority of poor survival-associated immune genes were positively correlated with the high expression of TFs in serous OC. The most critical TFs were CIITA, BATF, VDR, and CBX2. Among them, CIITA has been shown to drive MHC Class II expressing tumor cells as professional antigen presenting cell (APC) performers, thus activating the immune cells and constructing the specific optimal anti-tumor vaccine [13]. BATF can induce the T cell exhaustion during chronic infection, which is characterized by expression of inhibitory receptors and protect cells from excessive immunopathology [14, 15]. Besides, BATF inhibition can ameliorate the pathophysiologic responses in allergic asthma acting as the important transcription factor by regulating T and B-cell differentiation [16]. Vitamin D and the vitamin D receptor (VDR) is important in immunological regulation in disease such as inflammatory bowel diseases (IBD) and human immunodeficiency virus infection by modulating the function of monocytes/macrophages during infection [17, 18]. Furthermore, polycomb chromobox (CBX) proteins, especially CBX2 were down-regulated in macrophage upon viral infection. Cbx2 knockdown or silencing inhibited IFN-β production and played a critical role in antiviral innate immunity [19]. On the basis of the aforementioned findings, the specific TFs for promoters encoding may be readily translated to clinical practice.

Immune cells infiltration is the important features in tumor microenvironment (TME) of ovarian cancer. Early immune response is always presented with multiple types of immune cell infiltration and immunity-associated gene expression alteration. Previous studies showed that alternatively activated macrophages (M2) and neutrophils possess the pro-tumor roles and T follicular helper cells (Tfh) play an important role in immune cells recruitment. In our study, we showed that the genes in patients with high risk scores were correlated with enrichment in pro-tumor or anti-inflammatory pathways relating with M2 macrophages and neutrophils infiltration, while the genes in patients with low risk scores were correlated with enrichment in inflammatory pathways relating with M0 macrophages, Tfh cells and NK cells. The article reflected the landscape of immune infiltration in TME of ovarian cancer. The association between gene signature and immune cells infiltration would demonstrate the differences of immune cells infiltrates with different clinical prognosis, and it would be an attractive target for prediction of immune infiltration and therapy intervention.

Our study has some advantages. Firstly, we performed analysis with TCGA dataset which showed larger sample size, thus our gene signature was reliable and general. Secondly, the current study was based on the immune-related genes downloaded from the ImmPort database which showed a strong immune-based biological background, thus our study has the advantage on other models which screened from RNA-seq or the whole genome profiling, providing the novel immune landscape of microenvironment and immune-based biomarkers and targets for early diagnosis and molecule-targeted therapy exploration. Thirdly, our prognostic model had a promising survival prediction ability which was shown in ROC curves, and our signature simplified the complicated effects of immune genes in clinical outcomes and immunotherapy responses, making it easier for prognosis and therapy response prediction.

But our study also showed some limitation. First, we used the datasets from both GEO and TCGA to get more sufficient validation, and we downloaded the gene profiling information from GTEx dataset for the normal cases. Undoubtly it will show some statistic cohort bias and heterogeneity for the difference of platforms and differences in clinical care, clinical setting, and treatment. Second, only overall survival was remained to estimate the association between immune gene signature and clinical outcome to decrease the missing rate. This approach increased statistical power and data integrality, but it is also a limitation insofar as some patients’ information will be lost, and the signature will be more accurate if other survival parameters are included. Third, this study is developed with genes in ImmPort database, further biological experiments and validation are warranted in ovarian cancer. At the same time, the gene signature was validated in other two independent GEO datasets, but it will be more reliable with prospective cohort study in the future.

In summary, the current study constructed prognostic signature with the immune-related genes, providing a good ability for prognostic prediction. Network analysis revealed the regulatory relationship and the interaction between immune genes and transcription factors, providing the biomarkers for immunomodulators. Prospective and validation studies are necessary for further establishment of prediction accuracy with this gene signature. The network analysis is warranted to be validated to identify the critical role of transcription factors in survival outcome.

## Supplementary Information

**Additional file 1: Figure S1.** Study flowchart for profiling the immune gene-based signature with RNA-seq data. TGCA, The Cancer Genome Atlas; GTEx, Genotype-Tissue Expression; GEO, Gene Expression Omnibus; ROC, Receiver Operating Characteristic. **Figure S2.** Survival-associated immune genes in TCGA ovarian cancer cohorts. Unadjusted HRs (boxes) and 95% CI (horizontal lines) limited to DEIGs from TCGA dataset by using univariate COX analysis. TGCA, The Cancer Genome Atlas; DEIG, differential expression immune genes. **Figure S3.** LASSO COX regression to construct prognostic signature. LASSO, Least absolute shrinkage and selection operator. **Figure S4.** The correlation between gene signature and clinical features. Risk score correlated with age (A), and stage (B) in high grade serous ovarian cancer. **Figure S5.** The correlation between gene signature and immune cell. Risk score in the gene signature negatively correlated with Macrophages M0 (A), NK cells resting (B), and T cells follicular helper (C). Risk score in the gene signature positively correlated with Macrophages M2(D), Neutrophils(E), and T cells CD8(F). **Figure S6.** Biological function and GeneOntology analysis with 71 survival associated immune genes.

**Additional file 2: Appendix Table 1.** Clinical properties of the ovarian cancer patients used in the analysis.

**Additional file 3: Table S1.** Differentially expressed immune genes for serous ovarian cancer patients (*p*-value < 2.76 × 10^− 5^ and absolute fold change > 2). ConMean = Mean expression level of control group; treatMean = Mean expression level of treatment group; FC = fold change.

**Additional file 4: Table S2.** Prognostic immune genes for serous ovarian cancer patients. Index = risk score; HR, hazard ratio; HR.95 L, hazard ratio with lower 95% confidence index; HR.95H, hazard ratio with high 95% confidence index.

**Additional file 5: Table S3.** The correlation between gene signature and clinical features. *P* values were shown in ()

**Additional file 6: Table S4.** Differentially expressed transcription factors for serous ovarian cancer patients (*p-*value < 1.57 × 10^− 4^ and absolute fold change > 2). ConMean = Mean expression level of control group; treatMean = Mean expression level of treatment group; FC = fold change.

**Additional file 7: Table S5.** Immune related genes were identified in ChIP-seq data of CIITA, BATF, VDR, and CBX2 from ENCODE and GTRD databases.

## Data Availability

The datasets generated and analysed during the current study are available in the TCGA and GEO datasets that provide free online tools and resources.

## Abbreviations

OC: Ovarian cancer
TCGA: The Cancer Genome Atlas
TFs: Transcription factors
CTLA-4: Cytotoxic T lymphocyte-associated protein4
PD-1: Programmed cell death 1
NK: Natural killer
DEIGs: Differentially expressed immune gene
OS: Overall survival
LASSO: Least absolute shrinkage and selection operator
RS: Risk score
K-M: Kaplan-Meier
ROC: Receiver operating characteristic
AUC: Area under the curve
DETG: Differentially expressed transcription genes
DEGs: Differential expression genes
APC: Antigen presenting cell
VDR: Vitamin D receptor
IBD: Inflammatory bowel diseases
TME: Tumor microenvironment
