# Supplementary material for: Development of a multi-gene-based immune prognostic signature in ovarian Cancer
Source: J Ovarian Res. 2021 Jan 28;14:20. doi: 10.1186/s13048-021-00766-4 (PMC7844906; doi:10.1186/s13048-021-00766-4)
Supplement: Supplementary file 1 — Additional file 1: Figure S1. Study flowchart for profiling the immune gene-based signature with RNA-seq data. TGCA, The Cancer Genome Atlas; GTEx, Genotype-Tissue Expression; GEO, Gene Expression Omnibus; ROC, Receiver Operating Characteristic. Figure S2. Survival-associated immune genes in TCGA ovarian cancer cohorts. Unadjusted HRs (boxes) and 95% CI (horizontal lines) limited to DEIGs from TCGA dataset by using univariate COX analysis. TGCA, The Cancer Genome Atlas; DEIG, differential expression immune genes. Figure S3. LASSO COX regression to construct prognostic signature. LASSO, Least absolute shrinkage and selection operator. Figure S4. The correlation between gene signature and clinical features. Risk score correlated with age (A), and stage (B) in high grade serous ovarian cancer. Figure S5. The correlation between gene signature and immune cell. Risk score in the gene signature negatively correlated with Macrophages M0 (A), NK cells resting (B), and T cells follicular helper (C). Risk score in the gene signature positively correlated with Macrophages M2(D), Neutrophils(E), and T cells CD8(F). Figure S6. Biological function and GeneOntology analysis with 71 survival associated immune genes. [file 13048_2021_766_MOESM1_ESM.docx]

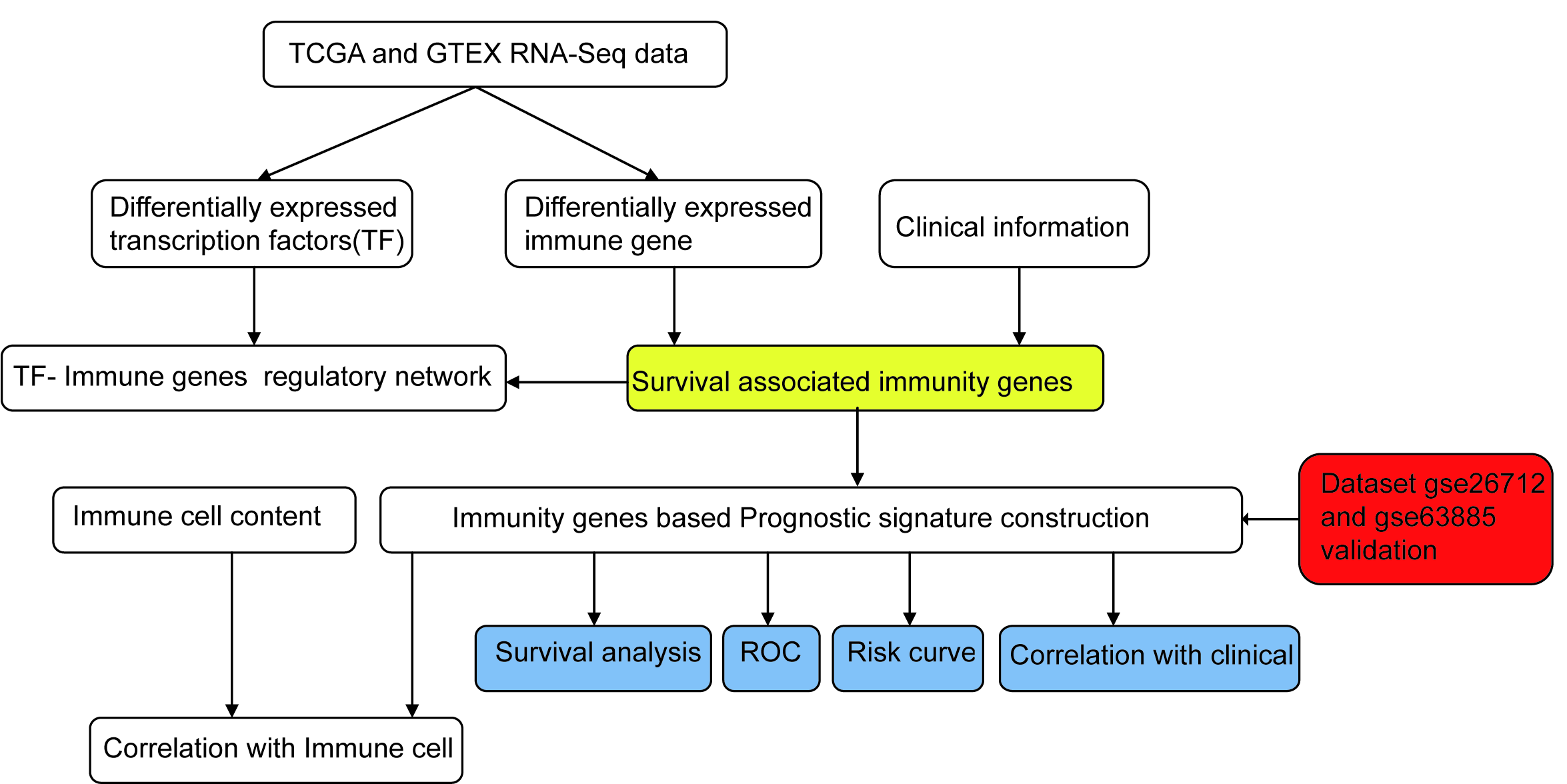


FigS1. Study flowchart for profiling the immune gene-based signature with RNA-seq data. TGCA, The Cancer Genome Atlas; GTEx, Genotype-Tissue Expression; GEO, Gene Expression Omnibus; ROC, Receiver Operating Characteristic.


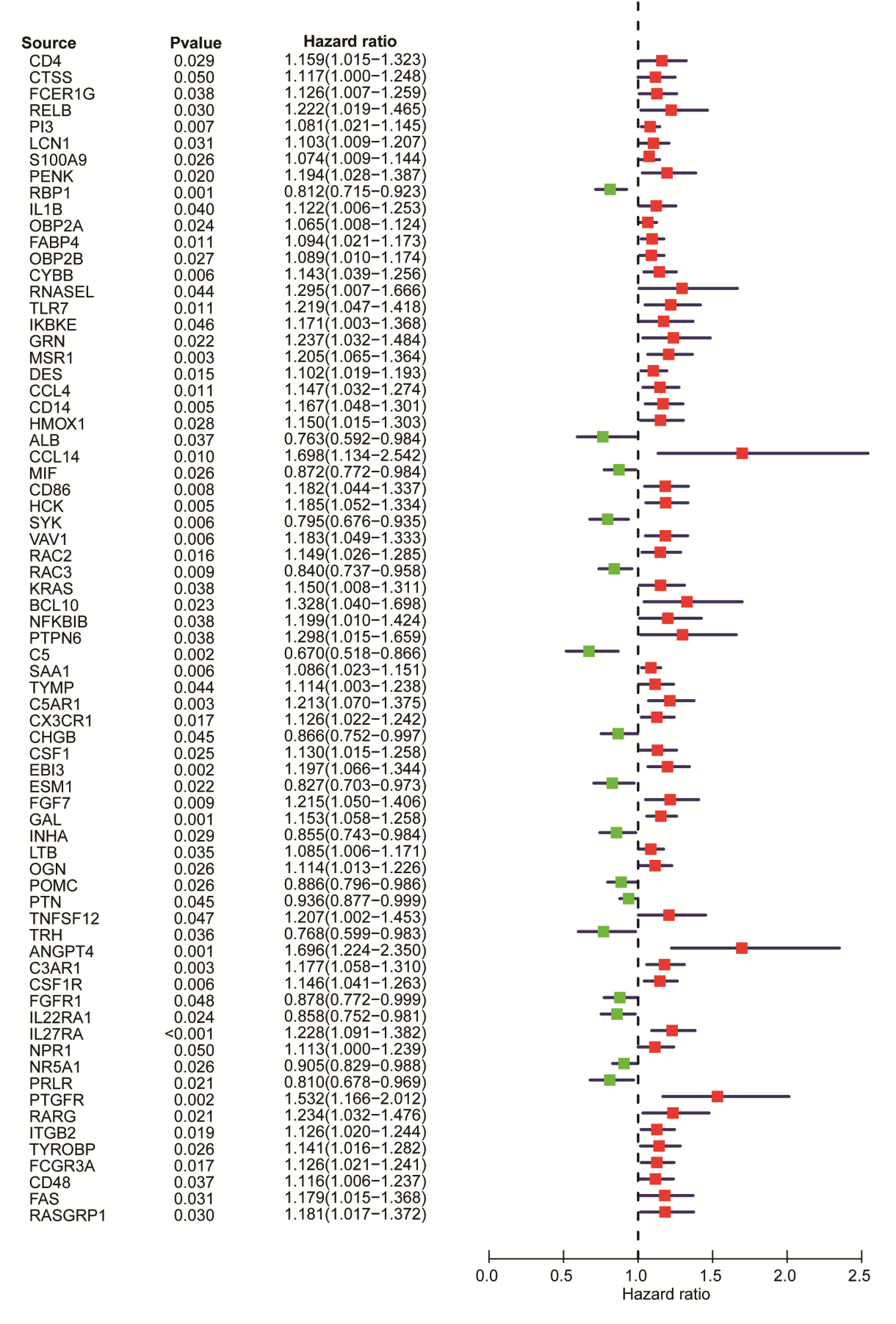


FigS2. Survival-associated immune genes in TCGA ovarian cancer cohorts. Unadjusted HRs (boxes) and 95% CI (horizontal lines) limited to DEIGs from TCGA dataset by using univariate COX analysis. TGCA, The Cancer Genome Atlas; DEIG, differential expression immune genes.


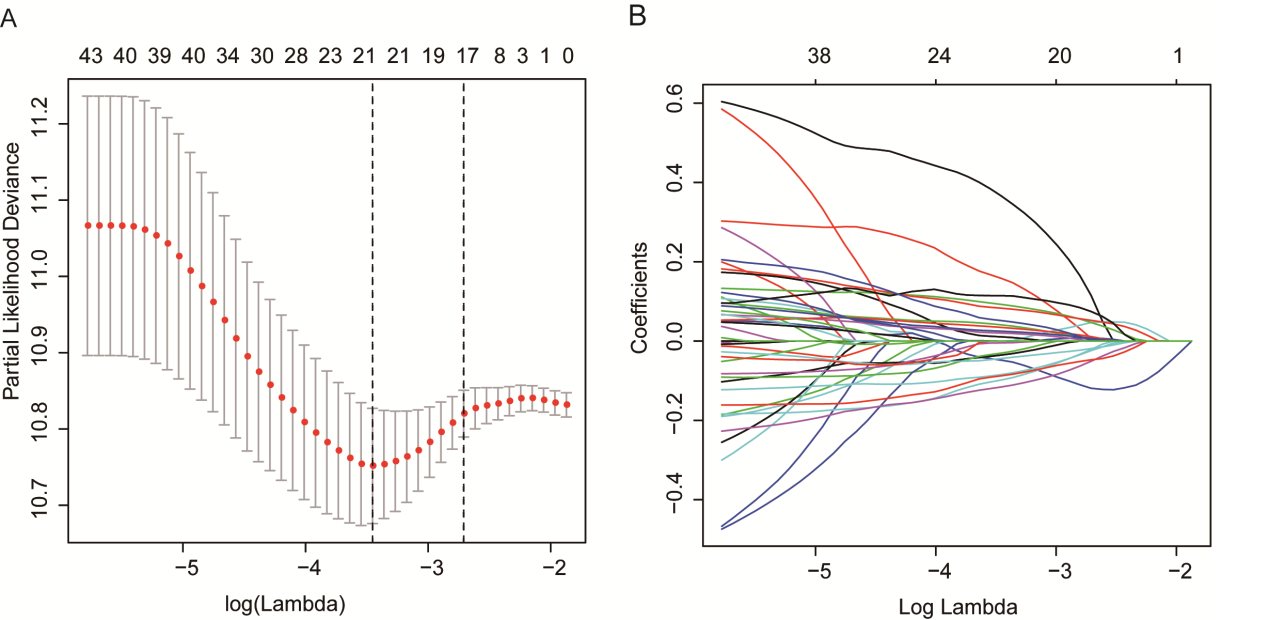


FigS3. LASSO COX regression to construct prognostic signature. LASSO, Least absolute shrinkage and selection operator.


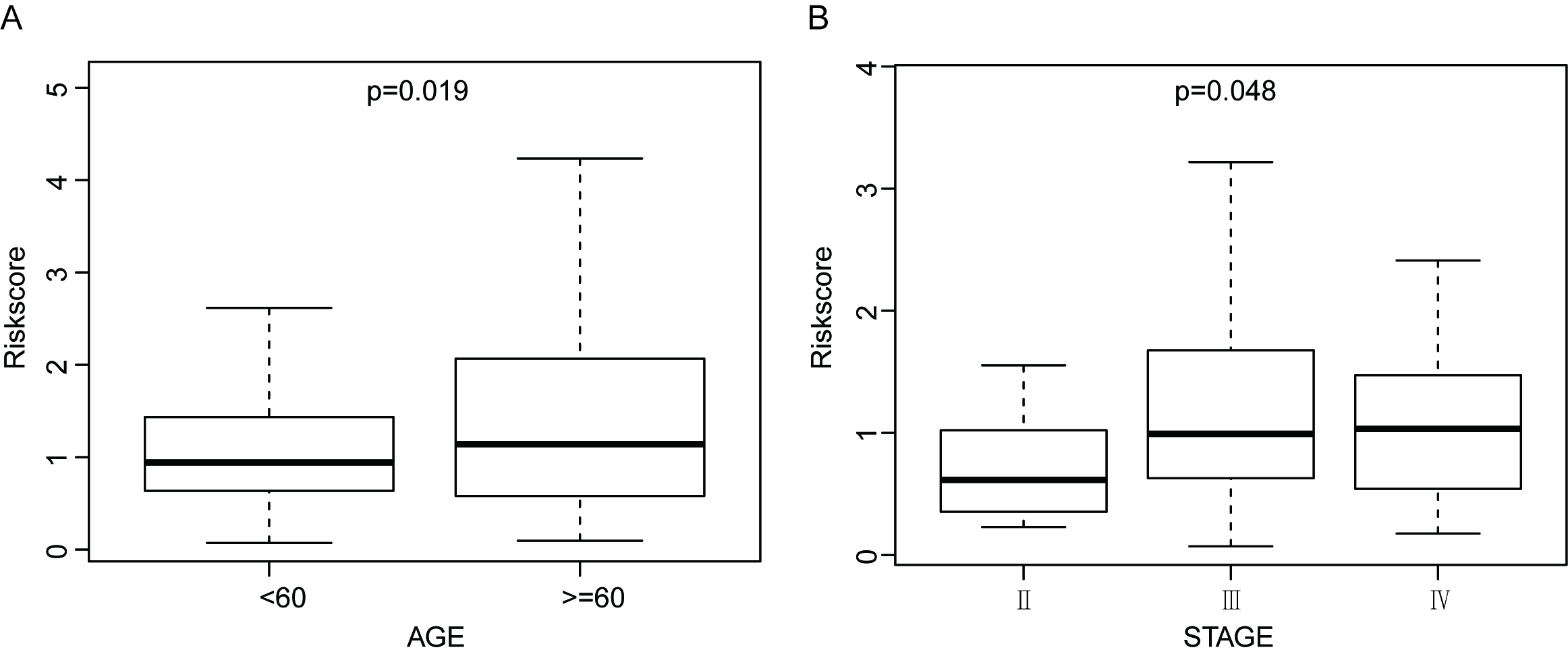


FigS4. The correlation between gene signature and clinical features. Risk score correlated with age (A), and stage (B) in high grade serous ovarian cancer.


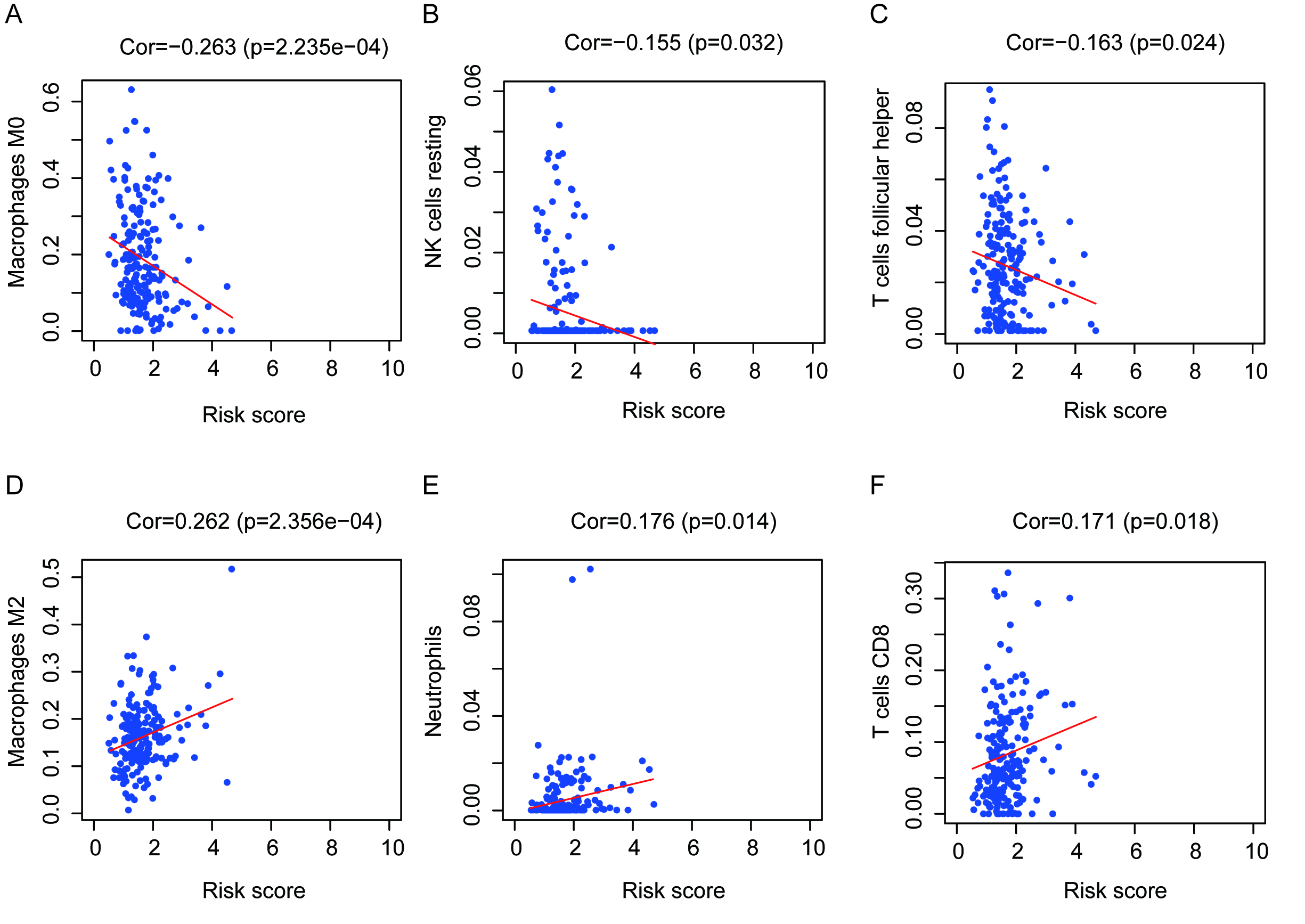


FigS5. The correlation between gene signature and immune cell. Risk score in the gene signature negatively correlated with Macrophages M0 (A), NK cells resting (B), and T cells follicular helper (C). Risk score in the gene signature positively correlated with Macrophages M2(D), Neutrophils(E), and T cells CD8(F).


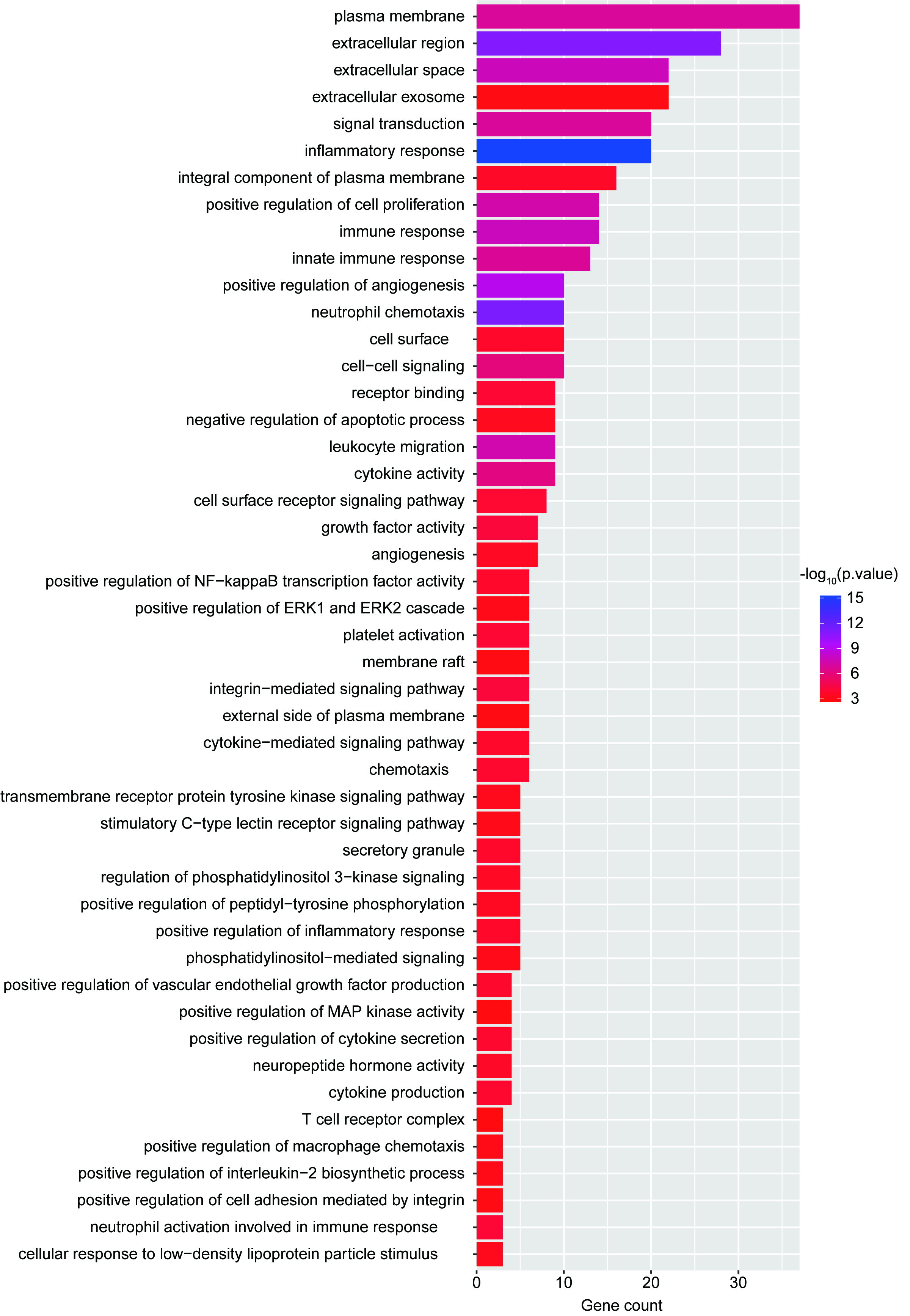


FigS6. Biological function and GeneOntology analysis with 71 survival associated immune genes
