# Supplementary material for: Development of a multi-gene-based immune prognostic signature in ovarian Cancer
Source: J Ovarian Res. 2021 Jan 28;14:20. doi: 10.1186/s13048-021-00766-4 (PMC7844906; doi:10.1186/s13048-021-00766-4)
Supplement: Supplementary file 2 — Additional file 2: Appendix Table 1. Clinical properties of the ovarian cancer patients used in the analysis. [file 13048_2021_766_MOESM2_ESM.docx]

TableS2. 21 hub genes for serous ovarian cancer patients.

| **Gene** | **Index** | **HR** | **HR.95L** | **HR.95H** | **P value** |
| --- | --- | --- | --- | --- | --- |
| IL27RA | 0.180863 | 1.19825 | 1.044462 | 1.374683 | 0.009861 |
| GAL | 0.145545 | 1.15667 | 1.039164 | 1.287464 | 0.007749 |
| RBP1 | -0.25545 | 0.774565 | 0.668142 | 0.89794 | 0.000705 |
| ANGPT4 | 0.437327 | 1.548563 | 1.012669 | 2.368046 | 0.043585 |
| EBI3 | 0.220041 | 1.246128 | 0.973519 | 1.595073 | 0.080654 |
| C5AR1 | 0.175933 | 1.192358 | 0.945735 | 1.503295 | 0.136734 |
| MSR1 | 0.389857 | 1.47677 | 1.041969 | 2.093009 | 0.028451 |
| HCK | -0.4864 | 0.614837 | 0.39204 | 0.964252 | 0.034131 |
| SYK | -0.20955 | 0.810948 | 0.674212 | 0.975415 | 0.026137 |
| CYBB | 0.740949 | 2.097926 | 1.367108 | 3.219419 | 0.000696 |
| PI3 | 0.11042 | 1.116747 | 1.040608 | 1.198458 | 0.002178 |
| CD86 | -0.89087 | 0.410298 | 0.243802 | 0.690496 | 0.000795 |
| FABP4 | 0.088639 | 1.092686 | 1.010441 | 1.181626 | 0.026411 |
| CX3CR1 | 0.159839 | 1.173322 | 1.009831 | 1.363282 | 0.036821 |
| ITGB2 | -0.42656 | 0.652751 | 0.479116 | 0.889313 | 0.006863 |
| PENK | 0.36938 | 1.446838 | 1.195653 | 1.750792 | 0.000147 |
| PRLR | -0.1746 | 0.839795 | 0.670296 | 1.052155 | 0.129026 |
| RARG | 0.194931 | 1.215227 | 0.969777 | 1.522801 | 0.090386 |
| ESM1 | -0.22681 | 0.797074 | 0.66332 | 0.957799 | 0.015519 |
| BCL10 | 0.231331 | 1.260276 | 0.94047 | 1.688832 | 0.121383 |
| OBP2A | 0.077202 | 1.08026 | 1.0164 | 1.148133 | 0.013021 |

Index=risk score; HR, hazard ratio; HR.95L, hazard ratio with lower 95% confidence index; HR.95H, hazard ratio with high 95% confidence index.
