# Supplementary material for: Development of a multi-gene-based immune prognostic signature in ovarian Cancer
Source: J Ovarian Res. 2021 Jan 28;14:20. doi: 10.1186/s13048-021-00766-4 (PMC7844906; doi:10.1186/s13048-021-00766-4)
Supplement: Supplementary file 5 — Additional file 5: Table S3. The correlation between gene signature and clinical features. P values were shown in () [file 13048_2021_766_MOESM5_ESM.docx]

TableS3. The correlation between gene signature and clinical features. P values were shown in ().

| Gene | OS（Days) | Survival status* | Grade | Age | Stage | Debulking status^#^ |
| --- | --- | --- | --- | --- | --- | --- |
| IL27RA | 292.745  (0.427) | -0.515  (0.607) | 0.635  (0.528) | -1.14  (0.255) | 0.576  (0.750) | -0.657  (0.512) |
| GAL | 283.492  (0.580) | 0.81  (0.419) | -0.559  (0.579) | 0.613  (0.540) | 0.555  (0.758) | -0.27  (0.788) |
| RBP1 | 286.328  (0.533) | 0.122  (0.903) | 0.449  (0.656) | 0.896  (0.371) | 0.364  (0.834) | -2.242  (0.026) |
| ANGPT4 | 299.839  (0.318) | -0.583  0.560) | -1.75  (0.082) | -1.509  (0.133) | 1.408  (0.495) | 0.708  (0.481) |
| EBI3 | 288.495  (0.497) | 1.797  (0.073) | -1.132  (0.263) | -0.66  (0.510) | 1.472  (0.479) | 0.62  (0.536) |
| C5AR1 | 288.347  (0.500) | -0.748  (0.455) | -1.958  (0.056) | -0.082  (0.935) | 0.058  (0.971) | 1.423  (0.157) |
| MSR1 | 282.42  (0.598) | 1.313  (0.190) | -1.23  (0.225) | -0.001  (1.000) | 0.033  (0.984) | 1.367  (0.173) |
| HCK | 280.37  (0.631) | 1.586  (0.114) | -1.179  (0.245) | -0.834  (0.405) | 2.565  (0.277) | 0.672  (0.502) |
| SYK | 292.268  (0.435) | -0.632  (0.528) | 0.444  (0.659) | 2.899  (0.004) | 0.144  (0.931) | -1.258  (0.211) |
| CYBB | 286.435  (0.532) | 1.396  (0.164) | -1.423  (0.162) | 0.122  (0.903) | 1.624  (0.444) | 0.86  (0.391) |
| PI3 | 297.478  (0.353) | -0.863  (0.389) | -0.865  (0.392) | -1.799  (0.073) | 0.222  (0.895) | 1.128  (0.261) |
| CD86 | 284.106  (0.570) | 1.718  (0.087) | -1.747  (0.087) | 0.331  (0.741) | 0.638  (0.727) | 1.12  (0.264) |
| FABP4 | 285.385  (0.549) | -0.934  (0.351) | -0.522  (0.604) | -1.583  (0.115) | 8.308  (0.016) | 2.408  (0.017) |
| CX3CR1 | 293.688  (0.412) | -1.334  (0.183) | -0.177  (0.860) | 0.782  (0.435) | 1.316  (0.518) | 0.248  (0.805) |
| ITGB2 | 292.453  (0.432) | 0.954  (0.341) | -0.638  (0.527) | 0.816  (0.415) | 1.676  (0.433) | 0.488  (0.626) |
| PENK | 286.231  (0.535) | -0.214  (0.830) | 0.837  (0.407) | -0.923  (0.357) | 1.178  (0.555) | -0.584  (0.560) |
| PRLR | 278.82  (0.656) | -0.075  (0.940) | 0.35  (0.728) | -0.532  (0.595) | 1.399  (0.497) | -0.26  (0.795) |
| RARG | 289.072  (0.488) | -1.699  (0.091) | -0.705  (0.484) | 1.001  (0.318) | 3.506  (0.173) | -0.462  (0.644) |
| ESM1 | 295.102  (0.390) | 1.531  (0.127) | 3.772  (4.298e-04) | 0.613  (0.540) | 13.886  (9.655e-04) | -1.537  (0.126) |
| BCL10 | 297.124  (0.358) | 1.062  (0.289) | 0.214  (0.831) | 3.432  (6.797e-04) | 2.131  (0.345) | -0.6  (0.549) |
| OBP2A | 290.971  (0.456) | -0.294  (0.769) | -0.329  (0.743) | 1.488  (0.138) | 0.223  (0.894) | -0.135  (0.893) |
| Riskscore | 284.685  (0.561) | -1.381  (0.169) | -1.524  (0.134) | -2.36  (0.019) | 6.066  (0.048) | 1.697  (0.092) |

*, 0 represents for alive while 1 is for dead. ^#^, 0 represents for sub-optimal debulking while 1 is for optimal debulking status.
