# Supplementary material for: Development of a multi-gene-based immune prognostic signature in ovarian Cancer
Source: J Ovarian Res. 2021 Jan 28;14:20. doi: 10.1186/s13048-021-00766-4 (PMC7844906; doi:10.1186/s13048-021-00766-4)
Supplement: Supplementary file 6 — Additional file 6: Table S4. Differentially expressed transcription factors for serous ovarian cancer patients (p-value < 1.57 × 10− 4 and absolute fold change > 2). ConMean = Mean expression level of control group; treatMean = Mean expression level of treatment group; FC = fold change. [file 13048_2021_766_MOESM6_ESM.docx]

TableS1. Differentially expressed transcription factors for serous ovarian cancer patients (p-value <1.57×10^-4^ and absolute fold change >2). ConMean=Mean expression level of control group; treatMean=Mean expression level of treatment group; FC=fold change.

| **ID** | **conMean** | **treatMean** | **logFC** | **pValue** | **fdr** |
| --- | --- | --- | --- | --- | --- |
| ARNTL | 4.945427 | 2.525462 | -2.41997 | 9.46E-48 | 1.08E-46 |
| ATF1 | 4.196231 | 5.353742 | 1.157511 | 6.76E-45 | 3.50E-44 |
| ATF7 | 4.268731 | 2.943206 | -1.32553 | 6.61E-48 | 8.45E-47 |
| BATF | 1.278149 | 2.763777 | 1.485628 | 1.84E-28 | 3.64E-28 |
| BCL11A | 1.16598 | 3.699611 | 2.533631 | 6.56E-40 | 2.09E-39 |
| BCL3 | 5.280694 | 6.597452 | 1.316758 | 1.49E-18 | 2.32E-18 |
| BRD2 | 7.885117 | 6.692721 | -1.1924 | 6.58E-46 | 4.06E-45 |
| BRF1 | 4.803591 | 2.497522 | -2.30607 | 6.20E-48 | 8.13E-47 |
| CBX2 | 2.221179 | 3.600238 | 1.379059 | 4.40E-27 | 8.38E-27 |
| CBX5 | 4.106593 | 5.271346 | 1.164753 | 1.40E-40 | 4.69E-40 |
| CBX7 | 6.229018 | 2.791919 | -3.4371 | 2.16E-48 | 5.79E-47 |
| CBX8 | 1.993515 | 3.203005 | 1.209489 | 4.46E-44 | 2.05E-43 |
| CDK9 | 7.28209 | 5.758695 | -1.52339 | 1.59E-46 | 1.14E-45 |
| CEBPA | 1.488667 | 4.046903 | 2.558236 | 5.36E-44 | 2.42E-43 |
| CEBPB | 6.555956 | 8.333757 | 1.7778 | 2.16E-43 | 9.21E-43 |
| CENPA | 0.793988 | 4.4728 | 3.678813 | 3.86E-48 | 6.32E-47 |
| CHD1 | 5.576325 | 3.006751 | -2.56957 | 2.14E-48 | 5.79E-47 |
| CHD2 | 5.951941 | 3.797606 | -2.15434 | 2.56E-48 | 5.79E-47 |
| CHD7 | 2.175132 | 3.580354 | 1.405222 | 6.95E-40 | 2.22E-39 |
| CIITA | 1.963738 | 3.238018 | 1.274281 | 1.19E-21 | 1.99E-21 |
| CUX1 | 5.790722 | 4.631889 | -1.15883 | 6.18E-46 | 3.83E-45 |
| E2F1 | 2.461286 | 5.579496 | 3.11821 | 7.52E-48 | 9.19E-47 |
| E2F3 | 1.685273 | 4.314897 | 2.629624 | 2.46E-48 | 5.79E-47 |
| E2F7 | 0.675648 | 2.12266 | 1.447012 | 1.44E-47 | 1.47E-46 |
| EBF1 | 3.387933 | 1.910445 | -1.47749 | 1.53E-34 | 3.71E-34 |
| EED | 4.65888 | 3.483296 | -1.17558 | 4.46E-40 | 1.44E-39 |
| EGR2 | 1.819108 | 3.194896 | 1.375788 | 6.64E-23 | 1.14E-22 |
| EHF | 0.837273 | 5.390054 | 4.552781 | 4.35E-46 | 2.77E-45 |
| ELL2 | 4.41377 | 2.542939 | -1.87083 | 2.15E-30 | 4.51E-30 |
| EP400 | 5.126603 | 3.572338 | -1.55426 | 1.10E-46 | 8.23E-46 |
| ESR1 | 3.274221 | 4.510635 | 1.236415 | 1.56E-28 | 3.09E-28 |
| ESRRA | 3.455923 | 5.139394 | 1.683471 | 2.07E-42 | 8.10E-42 |
| EZH1 | 6.386881 | 3.598866 | -2.78801 | 2.08E-48 | 5.79E-47 |
| EZH2 | 2.560422 | 4.334554 | 1.774132 | 3.92E-45 | 2.10E-44 |
| FOS | 8.460047 | 7.448542 | -1.01151 | 4.16E-08 | 5.16E-08 |
| FOSL1 | 1.449242 | 2.797121 | 1.34788 | 1.95E-27 | 3.76E-27 |
| FOXA2 | 0.47675 | 2.740008 | 2.263258 | 5.98E-43 | 2.46E-42 |
| FOXM1 | 1.641012 | 5.645853 | 4.00484 | 2.62E-48 | 5.79E-47 |
| FOXO1 | 5.824888 | 4.385479 | -1.43941 | 5.77E-37 | 1.56E-36 |
| FOXP1 | 4.876988 | 3.405242 | -1.47175 | 1.07E-45 | 6.32E-45 |
| FOXP2 | 2.776949 | 0.592233 | -2.18472 | 1.29E-47 | 1.36E-46 |
| FOXP3 | 0.896212 | 2.341119 | 1.444907 | 1.13E-43 | 4.95E-43 |
| GATA3 | 0.87227 | 2.122263 | 1.249994 | 8.89E-31 | 1.89E-30 |
| GATA4 | 6.628928 | 1.811204 | -4.81772 | 1.48E-45 | 8.48E-45 |
| GATA6 | 6.246276 | 4.761418 | -1.48486 | 1.04E-19 | 1.66E-19 |
| GREB1 | 7.760224 | 3.184464 | -4.57576 | 1.90E-45 | 1.07E-44 |
| GRHL2 | 0.710417 | 4.440736 | 3.73032 | 2.73E-47 | 2.50E-46 |
| GTF2I | 6.954786 | 4.493119 | -2.46167 | 2.37E-48 | 5.79E-47 |
| H2AFX | 4.5138 | 7.884667 | 3.370867 | 2.05E-48 | 5.79E-47 |
| HCFC1 | 4.807429 | 5.938601 | 1.131172 | 1.40E-39 | 4.38E-39 |
| HDAC2 | 6.003501 | 4.593203 | -1.4103 | 7.00E-43 | 2.85E-42 |
| HDAC3 | 6.2402 | 4.964573 | -1.27563 | 1.96E-47 | 1.88E-46 |
| HDAC6 | 6.812763 | 4.272982 | -2.53978 | 8.88E-48 | 1.04E-46 |
| HOXB7 | 1.180485 | 5.128499 | 3.948014 | 1.53E-46 | 1.10E-45 |
| IRF3 | 6.611322 | 5.520843 | -1.09048 | 3.22E-37 | 8.83E-37 |
| IRF5 | 1.849525 | 3.686724 | 1.837199 | 1.16E-41 | 4.21E-41 |
| JMJD1C | 5.517727 | 3.655844 | -1.86188 | 3.18E-47 | 2.82E-46 |
| JMJD6 | 5.325731 | 3.401717 | -1.92401 | 3.22E-48 | 5.94E-47 |
| KDM3A | 5.080805 | 4.043246 | -1.03756 | 1.71E-40 | 5.69E-40 |
| KDM4C | 3.912792 | 2.336877 | -1.57591 | 9.96E-48 | 1.12E-46 |
| KLF4 | 5.790626 | 3.541533 | -2.24909 | 1.43E-37 | 3.98E-37 |
| KLF5 | 2.429248 | 6.506164 | 4.076915 | 2.56E-46 | 1.73E-45 |
| LEF1 | 2.395213 | 4.082994 | 1.687781 | 1.65E-29 | 3.37E-29 |
| LIN9 | 2.130653 | 3.477267 | 1.346614 | 7.25E-47 | 5.72E-46 |
| LMNB1 | 2.398398 | 5.546363 | 3.147965 | 2.34E-48 | 5.79E-47 |
| MAF | 5.439192 | 3.761724 | -1.67747 | 8.39E-38 | 2.36E-37 |
| MAFF | 5.403698 | 3.750712 | -1.65299 | 7.22E-26 | 1.33E-25 |
| MAFK | 4.765497 | 3.671549 | -1.09395 | 5.69E-28 | 1.11E-27 |
| MBD3 | 6.084252 | 4.392609 | -1.69164 | 1.44E-47 | 1.47E-46 |
| MECP2 | 4.807857 | 3.691433 | -1.11642 | 2.12E-42 | 8.28E-42 |
| MEF2B | 2.517452 | 1.325728 | -1.19172 | 1.08E-41 | 3.92E-41 |
| MEF2C | 4.445858 | 2.341901 | -2.10396 | 6.72E-47 | 5.34E-46 |
| MITF | 3.723792 | 2.047317 | -1.67648 | 2.44E-44 | 1.17E-43 |
| MYB | 0.707887 | 2.794817 | 2.08693 | 4.65E-47 | 3.89E-46 |
| MYBL2 | 0.853286 | 6.442886 | 5.5896 | 2.19E-48 | 5.79E-47 |
| MYH11 | 7.188866 | 1.744021 | -5.44485 | 1.96E-47 | 1.88E-46 |
| NCAPG | 0.895766 | 3.53401 | 2.638244 | 5.32E-48 | 7.50E-47 |
| NFE2 | 0.787024 | 2.679481 | 1.892457 | 8.94E-41 | 3.04E-40 |
| NIPBL | 5.573973 | 4.127222 | -1.44675 | 9.46E-47 | 7.24E-46 |
| NOTCH1 | 2.81168 | 4.242005 | 1.430326 | 3.53E-30 | 7.36E-30 |
| NR2F1 | 6.427243 | 3.587723 | -2.83952 | 8.23E-44 | 3.65E-43 |
| NR2F2 | 8.057516 | 5.349398 | -2.70812 | 1.01E-46 | 7.64E-46 |
| NR4A1 | 9.828371 | 4.41978 | -5.40859 | 1.19E-47 | 1.28E-46 |
| OGT | 7.37179 | 5.480891 | -1.8909 | 1.52E-45 | 8.68E-45 |
| PBX3 | 6.292554 | 3.455191 | -2.83736 | 1.79E-47 | 1.75E-46 |
| POU5F1 | 1.06286 | 2.840691 | 1.777831 | 8.53E-37 | 2.29E-36 |
| PPARG | 4.077606 | 1.645504 | -2.4321 | 4.06E-45 | 2.17E-44 |
| RARG | 4.780423 | 5.910617 | 1.130194 | 7.40E-33 | 1.68E-32 |
| RBBP5 | 2.969073 | 4.364904 | 1.395831 | 6.78E-48 | 8.58E-47 |
| RBL2 | 5.84932 | 4.382799 | -1.46652 | 5.05E-46 | 3.18E-45 |
| RCOR1 | 3.586155 | 4.75261 | 1.166454 | 1.86E-42 | 7.30E-42 |
| RFX2 | 3.424208 | 2.41074 | -1.01347 | 2.61E-32 | 5.82E-32 |
| RFX5 | 3.985745 | 5.484268 | 1.498523 | 5.28E-47 | 4.36E-46 |
| RNF2 | 3.013865 | 4.88202 | 1.868155 | 2.56E-48 | 5.79E-47 |
| RUNX1T1 | 2.603412 | 1.070626 | -1.53279 | 1.07E-40 | 3.61E-40 |
| RXRA | 5.197437 | 3.940505 | -1.25693 | 1.66E-39 | 5.19E-39 |
| SALL4 | 0.83587 | 1.880389 | 1.044519 | 3.08E-25 | 5.60E-25 |
| SF1 | 8.49413 | 7.074654 | -1.41948 | 4.15E-47 | 3.54E-46 |
| SFMBT1 | 1.793615 | 3.471126 | 1.677511 | 3.65E-46 | 2.36E-45 |
| SFPQ | 8.75862 | 6.85009 | -1.90853 | 3.22E-48 | 5.94E-47 |
| SMAD2 | 5.055196 | 2.970429 | -2.08477 | 2.25E-48 | 5.79E-47 |
| SMAD4 | 5.647439 | 3.941961 | -1.70548 | 7.81E-48 | 9.49E-47 |
| SMARCC1 | 4.290374 | 6.051952 | 1.761578 | 1.06E-47 | 1.17E-46 |
| SOX17 | 2.346498 | 8.024702 | 5.678204 | 1.02E-47 | 1.14E-46 |
| SOX4 | 4.771141 | 6.83938 | 2.068239 | 3.35E-35 | 8.35E-35 |
| SOX9 | 0.877432 | 5.877604 | 5.000172 | 2.87E-48 | 5.81E-47 |
| SP2 | 3.933687 | 5.235285 | 1.301598 | 7.92E-47 | 6.19E-46 |
| SPDEF | 0.762494 | 4.501524 | 3.73903 | 7.65E-45 | 3.92E-44 |
| SREBF1 | 6.735193 | 4.935205 | -1.79999 | 2.12E-37 | 5.86E-37 |
| SRF | 4.876544 | 5.920502 | 1.043958 | 4.27E-33 | 9.79E-33 |
| STAT1 | 4.549819 | 6.992642 | 2.442823 | 1.68E-47 | 1.66E-46 |
| STAT2 | 7.226773 | 5.912272 | -1.3145 | 1.41E-39 | 4.43E-39 |
| STAT5B | 6.369548 | 4.078238 | -2.29131 | 2.49E-48 | 5.79E-47 |
| STAT6 | 7.915463 | 6.37106 | -1.5444 | 8.44E-47 | 6.53E-46 |
| SUMO1 | 5.817549 | 7.350069 | 1.53252 | 2.56E-48 | 5.79E-47 |
| TAF1 | 4.606192 | 3.372813 | -1.23338 | 4.46E-44 | 2.05E-43 |
| TAL1 | 1.787401 | 0.704237 | -1.08316 | 2.27E-45 | 1.26E-44 |
| TBL1XR1 | 4.662336 | 6.084307 | 1.42197 | 3.26E-46 | 2.13E-45 |
| TCF21 | 5.173478 | 1.668213 | -3.50526 | 7.64E-46 | 4.65E-45 |
| TCF7 | 1.993959 | 3.027879 | 1.03392 | 1.86E-28 | 3.67E-28 |
| TEAD4 | 2.495001 | 5.386436 | 2.891435 | 6.81E-47 | 5.40E-46 |
| TFAP2A | 1.066938 | 3.364891 | 2.297953 | 2.66E-31 | 5.72E-31 |
| TFAP2C | 0.974195 | 4.979076 | 4.004881 | 2.21E-45 | 1.23E-44 |
| TP53 | 4.728531 | 6.271002 | 1.542471 | 2.87E-19 | 4.54E-19 |
| USF2 | 7.799113 | 6.708053 | -1.09106 | 1.64E-39 | 5.14E-39 |
| VDR | 1.157648 | 3.614866 | 2.457218 | 2.53E-45 | 1.40E-44 |
| XBP1 | 6.411227 | 0.459283 | -5.95194 | 2.05E-48 | 5.79E-47 |
| XRN2 | 5.003792 | 6.633878 | 1.630085 | 9.34E-48 | 1.07E-46 |
| ZBTB17 | 5.441536 | 4.280702 | -1.16083 | 2.66E-44 | 1.27E-43 |
| ZBTB33 | 2.7498 | 4.871049 | 2.121249 | 2.05E-48 | 5.79E-47 |
