# Supplementary material for: Development of a multi-gene-based immune prognostic signature in ovarian Cancer
Source: J Ovarian Res. 2021 Jan 28;14:20. doi: 10.1186/s13048-021-00766-4 (PMC7844906; doi:10.1186/s13048-021-00766-4)
Supplement: Supplementary file 7 — Additional file 7: Table S5. Immune related genes were identified in ChIP-seq data of CIITA, BATF, VDR, and CBX2 from ENCODE and GTRD databases. [file 13048_2021_766_MOESM7_ESM.docx]

TableS5. Immune related genes were identified in ChIP-seq data of CIITA, BATF, VDR, and CBX2 from ENCODE and GTRD databases.

| **Genes** |
| --- |
| CTSS |
| FCER1G |
| RELB |
| S100A9 |
| IL1B |
| CYBB |
| IKBKE |
| MSR1 |
| CCL4 |
| HMOX1 |
| CD86 |
| HCK |
| VAV1 |
| RAC2 |
| CSF1 |
| EBI3 |
| C3AR1 |
| CSF1R |
| ITGB2 |
| TYROBP |
| CD48 |
| RASGRP1 |
| BCL10 |
| FGFR1 |
| FAS |
| PTGFR |
| NR5A1 |
| NFKBIB |
